# *catena*-Poly[[(2,2′-bipyridine)copper(II)]-μ-5-*tert*-butyl­isophthalato]

**DOI:** 10.1107/S1600536808035484

**Published:** 2008-11-08

**Authors:** Xiao-Ling Li, Miao-Ling Huang

**Affiliations:** a College of Chemistry and Chemical Engineering, Luoyang Normal University, Luoyang 471022, People’s Republic of China; b College of Chemistry and Chemical Engineering, Quanzhou Normal University, Quanzhou, Fujian 362000, People’s Republic of China

## Abstract

In the crystal structure of the title polymeric compound, [Cu(C_12_H_12_O_4_)(C_10_H_8_N_2_)]_*n*_, the asymmetric unit consists of one Cu^II^ ion, one 5-*tert*-butyl­isophthalate (tbip) and one 2,2′-bipyridine (bpy) ligand. The copper(II) ion is four-coordin­ated by two N atoms from bipy and two O atoms from two tbip ligands, leading to a distorted tetrahedral coordination. Each tbip ligand adopts a bis-monodentate coordination mode to connect two symmetry-related copper(II) ions, so forming a zigzag polymer chain parallel to [001]. The *tert*-butyl methyl groups are disordered over two positions with occupancies of 0.506 (6)/0.494 (6)

## Related literature

For related literature on the synthesis of flexible organic ligands, see: Chang *et al.* (2005 ▶); Ma, Chen *et al.* (2008 ▶); Xu *et al.* (2006 ▶). For related literature on coordination polymers, see: Ma, Wang, Huo *et al.* (2008 ▶); Ma, Wang, Wang *et al.* (2008 ▶); Pan *et al.* (2006 ▶); Yang *et al.* (2002 ▶). For bond-length data, see: Allen *et al.* (1987 ▶).
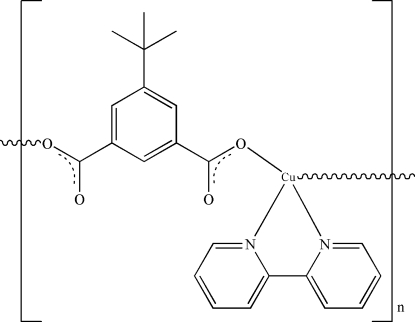


## Experimental

### 

#### Crystal data

[Cu(C_12_H_12_O_4_)(C_10_H_8_N_2_)]
*M* *_r_* = 439.94
Monoclinic, 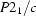

*a* = 8.905 (2) Å
*b* = 20.875 (5) Å
*c* = 11.564 (3) Å
β = 98.188 (3)°
*V* = 2127.8 (9) Å^3^
*Z* = 4
Mo *K*α radiation
μ = 1.06 mm^−1^
*T* = 296 (2) K
0.29 × 0.22 × 0.16 mm

#### Data collection

Bruker SMART CCD area-detector diffractometer
Absorption correction: multi-scan (*SADABS*; Bruker, 1997 ▶) *T*_min_ = 0.716, *T*_max_ = 0.845
15716 measured reflections
3949 independent reflections
3021 reflections with *I* > 2σ(*I*)
*R*_int_ = 0.040

#### Refinement

*R*[*F* ^2^ > 2σ(*F* ^2^)] = 0.045
*wR*(*F* ^2^) = 0.130
*S* = 1.05
3949 reflections
260 parameters
91 restraints
H-atom parameters constrained
Δρ_max_ = 0.64 e Å^−3^
Δρ_min_ = −0.55 e Å^−3^

### 

Data collection: *SMART* (Bruker, 1997 ▶); cell refinement: *SAINT* (Bruker, 1997 ▶); data reduction: *SAINT*; program(s) used to solve structure: *SHELXS97* (Sheldrick, 2008 ▶); program(s) used to refine structure: *SHELXL97* (Sheldrick, 2008 ▶); molecular graphics: *SHELXTL* (Sheldrick, 2008 ▶); software used to prepare material for publication: *SHELXTL*.

## Supplementary Material

Crystal structure: contains datablocks I, global. DOI: 10.1107/S1600536808035484/su2072sup1.cif

Structure factors: contains datablocks I. DOI: 10.1107/S1600536808035484/su2072Isup2.hkl

Additional supplementary materials:  crystallographic information; 3D view; checkCIF report

## Figures and Tables

**Table:** 

Cu1—O1	1.933 (2)
Cu1—O3^i^	1.956 (2)
Cu1—N1	1.985 (3)
Cu1—N2	1.983 (3)

**Table:** 

O1—Cu1—O3^i^	88.17 (11)
O1—Cu1—N1	94.83 (11)
O3^i^—Cu1—N1	172.79 (11)
O1—Cu1—N2	173.34 (11)
O3^i^—Cu1—N2	96.69 (11)
N1—Cu1—N2	80.88 (11)

Symmetry code: (i) 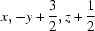.

**Fig. 1.**
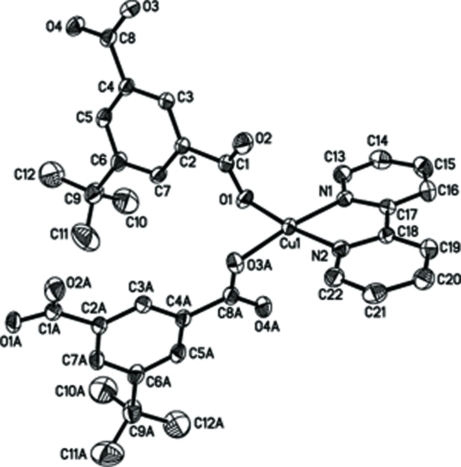
A view of the asymmetric unit of compound (I), with thermal ellipsoids drawn at the 50% probability level. H atoms have been omitted for clarity. Atoms with label A are related to those without by symmetry operation (x, -y+1.5, z+0.5).

**Fig. 2.**
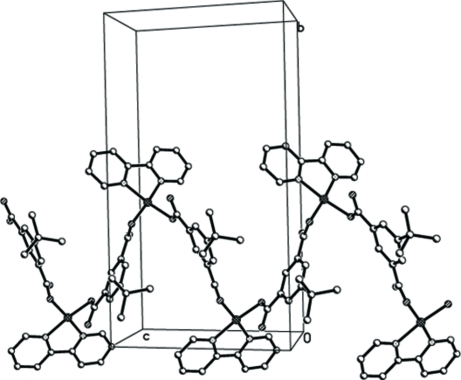
A partial view, along the a axis, of the crystal packing of compound (I) showing the zigzag polymer chain. H atoms have been omitted for clarity.

## supplementary crystallographic information

### Comment

It is well known that organic ligands play a crucial role in the design and construction of desirable frameworks. The changes in flexibility, length and symmetry of organic ligands can result in a remarkable class of materials bearing diverse architectures and functions. Thus, the construction of target molecules is a challenge for synthetic chemists (Ma *et al.*, 2008; Chang *et al.*,  2005; Xu *et al.*, 2006). Benzene-1,3-dicarboxylic acid (isophthalic acid, H2isop) and its derivatives, with special conformations such as, an angle of 120° between two carboxylic groups, present versatile coordination modes that can yield predetermined networks. Such ligands have been widely used to construct coordination polymers (Pan *et al.*, 2006; Yang *et al.*, 2002; Ma *et al.*, 2008).

The title compound, (I), was prepared by hydrothermal synthesis using 5-tert-butyl isophthalic acid, 2,2'-bipyridine and copper(II) actate. The asymmetric unit of (I) consists of one copper(II) ion, one tbip and one bipy ligand molecules (Fig. 1). Each copper(II) ion is four-coordinated by two nitrogen atoms from one bipy molecule and two oxygen atoms from two tbip ligands (Table 1). The coordination geometry of the copper(II) ion is distorted tetrahedral. The Cu—O bond lengths [1.933 (2)–1.965 (2) Å] are within the range reported for tetrahedral environments, and the Cu—N bond lengths [1.983 (3)–1.985 (3) Å] are also similar to those found in other tetrahedral copper complexes of bipy (Allen *et al.*, 1987). Each tbip ligand adopts the bis-monodentated coordination mode to connect two symmetry related copper(II) ions so forming a zigzag polymer chain (Fig. 2).

### Experimental

A mixture of 5-*tert*-butyl isophthalic acid (0.1 mmol, 23.1 mg), 2,2'-bipyridine (0.1 mmol, 15.8 mg), Cu(OAc).2.4H_2_O (0.05 mmol, 11.5 mg), NaOH (0.1 mmol, 4.0 mg) and H_2_O (15 ml) was placed in a Teflon-lined stainless steel vessel, and heated to 160 °C for 4 days. It was then cooled to room temperature over a period of 24 h. Blue block-like crystals of compound (I) were obtained.

### Refinement

The tertiary butyl methyl groups are disordered over two almost equally occupied positions: 0.506 (6)/0.494 (6). The H-atoms were included in calculated positions and treated as riding atoms: C—H = 0.93–0.96 Å with U_iso_(H) = 1.2 or 1.5U_eq_(parent C-atom).

### Crystal data

**Table:**

[Cu(C_12_H_12_O_4_)(C_10_H_8_N_2_)]	*F*_000_ = 908
*M**_r_* = 439.94	*D*_x_ = 1.373 Mg m^−^^3^
Monoclinic, *P*2_1_/*c*	Mo *K*α radiation λ = 0.71073 Å
Hall symbol: -P 2ybc	Cell parameters from 3455 reflections
*a* = 8.905 (2) Å	θ = 2.5–22.5º
*b* = 20.875 (5) Å	µ = 1.06 mm^−^^1^
*c* = 11.564 (3) Å	*T* = 296 (2) K
β = 98.188 (3)º	Block, blue
*V* = 2127.8 (9) Å^3^	0.29 × 0.22 × 0.16 mm
*Z* = 4	

### Data collection

**Table:**

Bruker SMART CCD area-detector diffractometer	3949 independent reflections
Radiation source: fine-focus sealed tube	3021 reflections with *I* > 2σ(*I*)
Monochromator: graphite	*R*_int_ = 0.040
*T* = 296(2) K	θ_max_ = 25.5º
φ and ω scans	θ_min_ = 2.5º
Absorption correction: multi-scan(SADABS; Bruker, 1997)	*h* = −10→10
*T*_min_ = 0.716, *T*_max_ = 0.845	*k* = −25→25
15716 measured reflections	*l* = −13→13

### Refinement

**Table:**

Refinement on *F*^2^	Secondary atom site location: difference Fourier map
Least-squares matrix: full	Hydrogen site location: inferred from neighbouring sites
*R*[*F*^2^ > 2σ(*F*^2^)] = 0.045	H-atom parameters constrained
*wR*(*F*^2^) = 0.130	*w* = 1/[σ^2^(*F*_o_^2^) + (0.065*P*)^2^ + 1.7017*P*] where *P* = (*F*_o_^2^ + 2*F*_c_^2^)/3
*S* = 1.05	(Δ/σ)_max_ = 0.001
3949 reflections	Δρ_max_ = 0.64 e Å^−^^3^
260 parameters	Δρ_min_ = −0.54 e Å^−^^3^
91 restraints	Extinction correction: none
Primary atom site location: structure-invariant direct methods	

### Special details

**Table:**

Geometry. All esds (except the esd in the dihedral angle between two l.s. planes) are estimated using the full covariance matrix. The cell esds are taken into account individually in the estimation of esds in distances, angles and torsion angles; correlations between esds in cell parameters are only used when they are defined by crystal symmetry. An approximate (isotropic) treatment of cell esds is used for estimating esds involving l.s. planes.
Refinement. Refinement of F^2^ against ALL reflections. The weighted R-factor wR and goodness of fit S are based on F^2^, conventional R-factors R are based on F, with F set to zero for negative F^2^. The threshold expression of F^2^ > 2sigma(F^2^) is used only for calculating R-factors(gt) etc. and is not relevant to the choice of reflections for refinement. R-factors based on F^2^ are statistically about twice as large as those based on F, and R- factors based on ALL data will be even larger.

### Fractional atomic coordinates and isotropic or equivalent isotropic displacement parameters (Å^2^)

**Table:**

	*x*	*y*	*z*	*U*_iso_*/*U*_eq_	Occ. (<1)
C10	−0.2988 (8)	0.7849 (4)	0.0801 (8)	0.0991 (17)	0.50
H10A	−0.3808	0.7876	0.1255	0.149*	0.50
H10B	−0.2580	0.7422	0.0851	0.149*	0.50
H10C	−0.3355	0.7948	0.0000	0.149*	0.50
C11	−0.1382 (9)	0.8222 (4)	0.2581 (6)	0.1033 (17)	0.50
H11A	−0.0577	0.8506	0.2893	0.155*	0.50
H11B	−0.1070	0.7786	0.2736	0.155*	0.50
H11C	−0.2266	0.8310	0.2943	0.155*	0.50
C12	−0.2268 (8)	0.9003 (3)	0.1063 (7)	0.1019 (19)	0.50
H12A	−0.3113	0.9085	0.1473	0.153*	0.50
H12B	−0.2570	0.9071	0.0242	0.153*	0.50
H12C	−0.1450	0.9288	0.1341	0.153*	0.50
C10'	−0.2432 (9)	0.7777 (3)	0.1868 (8)	0.0991 (17)	0.50
H10D	−0.2073	0.7791	0.2691	0.149*	0.50
H10E	−0.2138	0.7378	0.1553	0.149*	0.50
H10F	−0.3518	0.7813	0.1743	0.149*	0.50
C11'	−0.1082 (9)	0.8784 (4)	0.2317 (7)	0.1033 (17)	0.50
H11D	−0.0967	0.9208	0.2021	0.155*	0.50
H11E	−0.0112	0.8626	0.2671	0.155*	0.50
H11F	−0.1764	0.8796	0.2890	0.155*	0.50
C12'	−0.2878 (8)	0.8749 (4)	0.0548 (7)	0.1019 (19)	0.50
H12D	−0.3658	0.8871	0.0998	0.153*	0.50
H12E	−0.3324	0.8523	−0.0138	0.153*	0.50
H12F	−0.2371	0.9125	0.0323	0.153*	0.50
Cu1	0.30126 (5)	0.570966 (18)	0.17572 (3)	0.03784 (16)	
O1	0.1606 (3)	0.63474 (11)	0.1038 (2)	0.0497 (6)	
O2	0.3724 (3)	0.67027 (13)	0.0490 (3)	0.0598 (7)	
O3	0.3259 (3)	0.87708 (12)	−0.1821 (2)	0.0507 (6)	
O4	0.1347 (3)	0.94173 (12)	−0.1635 (2)	0.0542 (7)	
N1	0.2646 (3)	0.50997 (13)	0.0430 (2)	0.0368 (6)	
N2	0.4556 (3)	0.50539 (13)	0.2322 (2)	0.0371 (6)	
C1	0.2394 (5)	0.67826 (18)	0.0641 (3)	0.0510 (8)	
C2	0.1631 (4)	0.74186 (15)	0.0367 (3)	0.0397 (8)	
C3	0.2214 (4)	0.78533 (16)	−0.0351 (3)	0.0394 (8)	
H3	0.3084	0.7756	−0.0676	0.047*	
C4	0.1487 (4)	0.84380 (15)	−0.0584 (3)	0.0378 (8)	
C5	0.0213 (4)	0.85804 (16)	−0.0065 (3)	0.0430 (8)	
H5	−0.0261	0.8974	−0.0220	0.052*	
C6	−0.0377 (4)	0.81567 (17)	0.0678 (3)	0.0460 (9)	
C7	0.0343 (4)	0.75671 (16)	0.0860 (3)	0.0448 (9)	
H7	−0.0050	0.7264	0.1325	0.054*	
C8	0.2057 (4)	0.89150 (16)	−0.1391 (3)	0.0422 (8)	
C9	−0.1753 (6)	0.8323 (2)	0.1271 (5)	0.0809 (12)	
C13	0.1605 (4)	0.51736 (18)	−0.0526 (3)	0.0469 (9)	
H13	0.1031	0.5547	−0.0608	0.056*	
C14	0.1365 (4)	0.4717 (2)	−0.1382 (3)	0.0541 (10)	
H14	0.0625	0.4775	−0.2026	0.065*	
C15	0.2233 (5)	0.4176 (2)	−0.1271 (4)	0.0575 (11)	
H15	0.2087	0.3860	−0.1842	0.069*	
C16	0.3328 (4)	0.40997 (18)	−0.0308 (3)	0.0485 (9)	
H16	0.3938	0.3736	−0.0230	0.058*	
C17	0.3503 (4)	0.45690 (16)	0.0533 (3)	0.0352 (7)	
C18	0.4614 (4)	0.45458 (16)	0.1610 (3)	0.0353 (7)	
C19	0.5640 (4)	0.40508 (18)	0.1901 (3)	0.0478 (9)	
H19	0.5675	0.3703	0.1402	0.057*	
C20	0.6603 (5)	0.4085 (2)	0.2941 (4)	0.0556 (10)	
H20	0.7289	0.3755	0.3156	0.067*	
C21	0.6549 (5)	0.4606 (2)	0.3664 (3)	0.0548 (10)	
H21	0.7203	0.4638	0.4364	0.066*	
C22	0.5505 (4)	0.50766 (18)	0.3323 (3)	0.0475 (9)	
H22	0.5457	0.5428	0.3811	0.057*	

### Atomic displacement parameters (Å^2^)

**Table:**

	*U*^11^	*U*^22^	*U*^33^	*U*^12^	*U*^13^	*U*^23^
C10	0.081 (4)	0.096 (3)	0.134 (4)	0.009 (3)	0.061 (3)	0.009 (3)
C11	0.101 (4)	0.102 (4)	0.118 (4)	0.020 (3)	0.058 (3)	−0.004 (3)
C12	0.087 (4)	0.084 (3)	0.144 (5)	0.035 (3)	0.048 (3)	0.007 (3)
C10'	0.081 (4)	0.096 (3)	0.134 (4)	0.009 (3)	0.061 (3)	0.009 (3)
C11'	0.101 (4)	0.102 (4)	0.118 (4)	0.020 (3)	0.058 (3)	−0.004 (3)
C12'	0.087 (4)	0.084 (3)	0.144 (5)	0.035 (3)	0.048 (3)	0.007 (3)
Cu1	0.0495 (3)	0.0268 (2)	0.0376 (3)	0.00568 (18)	0.00751 (18)	0.00124 (16)
O1	0.0618 (16)	0.0312 (13)	0.0578 (16)	0.0093 (12)	0.0143 (13)	0.0113 (11)
O2	0.0639 (14)	0.0477 (13)	0.0722 (16)	0.0222 (12)	0.0247 (13)	0.0112 (12)
O3	0.0599 (17)	0.0424 (14)	0.0514 (16)	−0.0017 (12)	0.0132 (13)	0.0113 (12)
O4	0.0725 (18)	0.0373 (14)	0.0517 (16)	0.0066 (13)	0.0042 (13)	0.0124 (12)
N1	0.0414 (16)	0.0347 (15)	0.0340 (15)	0.0035 (12)	0.0042 (12)	0.0052 (12)
N2	0.0448 (16)	0.0336 (15)	0.0322 (14)	−0.0010 (12)	0.0029 (12)	0.0018 (12)
C1	0.0611 (16)	0.0381 (16)	0.0573 (18)	0.0190 (15)	0.0207 (15)	0.0079 (14)
C2	0.051 (2)	0.0288 (17)	0.0407 (19)	0.0085 (15)	0.0102 (16)	0.0041 (14)
C3	0.046 (2)	0.0351 (18)	0.0373 (18)	0.0047 (15)	0.0085 (15)	0.0008 (15)
C4	0.046 (2)	0.0293 (16)	0.0363 (18)	0.0006 (14)	0.0008 (15)	0.0015 (14)
C5	0.046 (2)	0.0299 (18)	0.051 (2)	0.0079 (15)	−0.0001 (16)	0.0030 (15)
C6	0.047 (2)	0.0335 (18)	0.059 (2)	0.0083 (16)	0.0106 (17)	0.0025 (17)
C7	0.051 (2)	0.0324 (18)	0.055 (2)	0.0062 (16)	0.0187 (17)	0.0105 (16)
C8	0.058 (2)	0.0337 (18)	0.0317 (17)	−0.0047 (17)	−0.0048 (16)	0.0022 (14)
C9	0.075 (3)	0.062 (2)	0.118 (3)	0.021 (2)	0.054 (3)	0.011 (2)
C13	0.047 (2)	0.049 (2)	0.043 (2)	0.0062 (17)	0.0025 (16)	0.0034 (17)
C14	0.047 (2)	0.070 (3)	0.043 (2)	−0.009 (2)	−0.0010 (17)	−0.0015 (19)
C15	0.062 (3)	0.060 (3)	0.049 (2)	−0.009 (2)	0.0051 (19)	−0.020 (2)
C16	0.052 (2)	0.042 (2)	0.052 (2)	0.0024 (17)	0.0075 (18)	−0.0099 (17)
C17	0.0374 (18)	0.0351 (17)	0.0343 (17)	−0.0003 (14)	0.0090 (14)	0.0010 (14)
C18	0.0388 (18)	0.0322 (17)	0.0367 (18)	0.0022 (14)	0.0112 (14)	0.0039 (14)
C19	0.053 (2)	0.043 (2)	0.048 (2)	0.0117 (18)	0.0132 (17)	0.0019 (17)
C20	0.055 (2)	0.058 (2)	0.053 (2)	0.020 (2)	0.0061 (19)	0.012 (2)
C21	0.057 (2)	0.061 (3)	0.044 (2)	0.004 (2)	−0.0044 (18)	0.0116 (19)
C22	0.057 (2)	0.043 (2)	0.041 (2)	−0.0016 (18)	0.0012 (17)	0.0005 (16)

### Geometric parameters (Å, °)

**Table:**

C10—C9	1.521 (9)	N1—C17	1.341 (4)
C10—H10A	0.9600	N1—C13	1.347 (4)
C10—H10B	0.9600	N2—C22	1.333 (4)
C10—H10C	0.9600	N2—C18	1.348 (4)
C11—C9	1.519 (9)	C1—C2	1.505 (5)
C11—H11A	0.9600	C2—C3	1.381 (5)
C11—H11B	0.9600	C2—C7	1.386 (5)
C11—H11C	0.9600	C3—C4	1.390 (5)
C12—C9	1.501 (7)	C3—H3	0.9300
C12—H12A	0.9600	C4—C5	1.389 (5)
C12—H12B	0.9600	C4—C8	1.502 (5)
C12—H12C	0.9600	C5—C6	1.387 (5)
C10'—C9	1.504 (8)	C5—H5	0.9300
C10'—H10D	0.9600	C6—C7	1.390 (5)
C10'—H10E	0.9600	C6—C9	1.527 (6)
C10'—H10F	0.9600	C7—H7	0.9300
C11'—C9	1.595 (9)	C8—Cu1^ii^	2.538 (4)
C11'—H11D	0.9600	C13—C14	1.368 (5)
C11'—H11E	0.9600	C13—H13	0.9300
C11'—H11F	0.9600	C14—C15	1.365 (6)
C12'—C9	1.502 (8)	C14—H14	0.9300
C12'—H12D	0.9600	C15—C16	1.382 (6)
C12'—H12E	0.9600	C15—H15	0.9300
C12'—H12F	0.9600	C16—C17	1.373 (5)
Cu1—O1	1.933 (2)	C16—H16	0.9300
Cu1—O3^i^	1.956 (2)	C17—C18	1.477 (5)
Cu1—N1	1.985 (3)	C18—C19	1.388 (5)
Cu1—N2	1.983 (3)	C19—C20	1.376 (5)
Cu1—C8^i^	2.538 (4)	C19—H19	0.9300
O1—C1	1.273 (4)	C20—C21	1.377 (6)
O2—C1	1.233 (5)	C20—H20	0.9300
O3—C8	1.279 (5)	C21—C22	1.372 (5)
O3—Cu1^ii^	1.956 (2)	C21—H21	0.9300
O4—C8	1.236 (4)	C22—H22	0.9300
			
C9—C10—H10A	109.5	C6—C5—C4	122.4 (3)
C9—C10—H10B	109.5	C6—C5—H5	118.8
H10A—C10—H10B	109.5	C4—C5—H5	118.8
C9—C10—H10C	109.5	C5—C6—C7	116.8 (3)
H10A—C10—H10C	109.5	C5—C6—C9	122.1 (3)
H10B—C10—H10C	109.5	C7—C6—C9	121.0 (4)
C9—C11—H11A	109.5	C2—C7—C6	121.8 (3)
C9—C11—H11B	109.5	C2—C7—H7	119.1
H11A—C11—H11B	109.5	C6—C7—H7	119.1
C9—C11—H11C	109.5	O4—C8—O3	122.7 (3)
H11A—C11—H11C	109.5	O4—C8—C4	119.8 (4)
H11B—C11—H11C	109.5	O3—C8—C4	117.5 (3)
C9—C12—H12A	109.5	O4—C8—Cu1^ii^	76.6 (2)
C9—C12—H12B	109.5	O3—C8—Cu1^ii^	49.07 (17)
H12A—C12—H12B	109.5	C4—C8—Cu1^ii^	155.9 (2)
C9—C12—H12C	109.5	C12—C9—C10'	131.1 (4)
H12A—C12—H12C	109.5	C12'—C9—C10'	115.1 (5)
H12B—C12—H12C	109.5	C12—C9—C11	108.0 (5)
C9—C10'—H10D	109.5	C12'—C9—C11	132.1 (5)
C9—C10'—H10E	109.5	C10'—C9—C11	58.7 (3)
H10D—C10'—H10E	109.5	C12—C9—C10	111.7 (5)
C9—C10'—H10F	109.5	C12'—C9—C10	78.2 (4)
H10D—C10'—H10F	109.5	C10'—C9—C10	49.7 (2)
H10E—C10'—H10F	109.5	C11—C9—C10	108.0 (4)
C9—C11'—H11D	109.5	C12—C9—C6	112.9 (4)
C9—C11'—H11E	109.5	C12'—C9—C6	113.5 (5)
H11D—C11'—H11E	109.5	C10'—C9—C6	115.8 (4)
C9—C11'—H11F	109.5	C11—C9—C6	110.0 (5)
H11D—C11'—H11F	109.5	C10—C9—C6	106.1 (5)
H11E—C11'—H11F	109.5	C12—C9—C11'	67.9 (3)
C9—C12'—H12D	109.5	C12'—C9—C11'	102.3 (4)
C9—C12'—H12E	109.5	C10'—C9—C11'	103.9 (5)
H12D—C12'—H12E	109.5	C11—C9—C11'	47.3 (2)
C9—C12'—H12F	109.5	C10—C9—C11'	146.9 (5)
H12D—C12'—H12F	109.5	C6—C9—C11'	103.9 (5)
H12E—C12'—H12F	109.5	N1—C13—C14	122.2 (4)
O1—Cu1—O3^i^	88.17 (11)	N1—C13—H13	118.9
O1—Cu1—N1	94.83 (11)	C14—C13—H13	118.9
O3^i^—Cu1—N1	172.79 (11)	C15—C14—C13	118.7 (4)
O1—Cu1—N2	173.34 (11)	C15—C14—H14	120.6
O3^i^—Cu1—N2	96.69 (11)	C13—C14—H14	120.6
N1—Cu1—N2	80.88 (11)	C14—C15—C16	119.8 (4)
O1—Cu1—C8^i^	82.87 (11)	C14—C15—H15	120.1
O3^i^—Cu1—C8^i^	29.60 (11)	C16—C15—H15	120.1
N2—Cu1—C8^i^	103.60 (11)	C17—C16—C15	119.0 (4)
N1—Cu1—C8^i^	144.33 (12)	C17—C16—H16	120.5
C1—O1—Cu1	106.8 (2)	C15—C16—H16	120.5
C8—O3—Cu1^ii^	101.3 (2)	N1—C17—C16	121.4 (3)
C17—N1—C13	118.9 (3)	N1—C17—C18	114.0 (3)
C17—N1—Cu1	115.6 (2)	C16—C17—C18	124.6 (3)
C13—N1—Cu1	125.4 (2)	N2—C18—C19	121.3 (3)
C22—N2—C18	118.9 (3)	N2—C18—C17	114.2 (3)
C22—N2—Cu1	125.8 (2)	C19—C18—C17	124.5 (3)
C18—N2—Cu1	115.3 (2)	C20—C19—C18	118.7 (4)
O2—C1—O1	123.0 (3)	C20—C19—H19	120.7
O2—C1—C2	120.3 (3)	C18—C19—H19	120.7
O1—C1—C2	116.7 (3)	C19—C20—C21	120.0 (4)
C3—C2—C7	120.3 (3)	C19—C20—H20	120.0
C3—C2—C1	120.6 (3)	C21—C20—H20	120.0
C7—C2—C1	119.1 (3)	C22—C21—C20	118.2 (4)
C2—C3—C4	119.3 (3)	C22—C21—H21	120.9
C2—C3—H3	120.4	C20—C21—H21	120.9
C4—C3—H3	120.4	N2—C22—C21	123.0 (4)
C5—C4—C3	119.4 (3)	N2—C22—H22	118.5
C5—C4—C8	119.7 (3)	C21—C22—H22	118.5
C3—C4—C8	120.9 (3)		
			
O3^i^—Cu1—O1—C1	84.9 (3)	C3—C4—C8—Cu1^ii^	47.6 (8)
N1—Cu1—O1—C1	−101.7 (3)	C5—C6—C9—C12	−6.0 (7)
C8^i^—Cu1—O1—C1	114.1 (3)	C7—C6—C9—C12	174.1 (5)
O1—Cu1—N1—C17	176.4 (2)	C5—C6—C9—C12'	32.7 (7)
N2—Cu1—N1—C17	1.5 (2)	C7—C6—C9—C12'	−147.2 (5)
C8^i^—Cu1—N1—C17	−99.1 (3)	C5—C6—C9—C10'	169.2 (6)
N2—Cu1—N1—C13	−180.0 (3)	C7—C6—C9—C10'	−10.7 (8)
C8^i^—Cu1—N1—C13	79.4 (3)	C5—C6—C9—C11	−126.8 (5)
O3^i^—Cu1—N2—C22	−7.2 (3)	C7—C6—C9—C11	53.3 (7)
N1—Cu1—N2—C22	179.6 (3)	C5—C6—C9—C10	116.6 (5)
C8^i^—Cu1—N2—C22	−36.5 (3)	C7—C6—C9—C10	−63.3 (6)
O3^i^—Cu1—N2—C18	172.5 (2)	C5—C6—C9—C11'	−77.6 (6)
N1—Cu1—N2—C18	−0.6 (2)	C7—C6—C9—C11'	102.6 (5)
C8^i^—Cu1—N2—C18	143.3 (2)	C17—N1—C13—C14	1.8 (5)
Cu1—O1—C1—O2	17.2 (5)	Cu1—N1—C13—C14	−176.7 (3)
Cu1—O1—C1—C2	−162.2 (3)	N1—C13—C14—C15	−1.4 (6)
O2—C1—C2—C3	18.7 (6)	C13—C14—C15—C16	−0.1 (6)
O1—C1—C2—C3	−162.0 (3)	C14—C15—C16—C17	1.1 (6)
O2—C1—C2—C7	−160.4 (4)	C13—N1—C17—C16	−0.7 (5)
O1—C1—C2—C7	19.0 (5)	Cu1—N1—C17—C16	178.0 (3)
C7—C2—C3—C4	−0.7 (5)	C13—N1—C17—C18	179.3 (3)
C1—C2—C3—C4	−179.7 (3)	Cu1—N1—C17—C18	−2.1 (4)
C2—C3—C4—C5	1.7 (5)	C15—C16—C17—N1	−0.7 (6)
C2—C3—C4—C8	−178.1 (3)	C15—C16—C17—C18	179.3 (3)
C3—C4—C5—C6	−0.5 (5)	C22—N2—C18—C19	−0.1 (5)
C8—C4—C5—C6	179.3 (3)	Cu1—N2—C18—C19	−179.8 (3)
C4—C5—C6—C7	−1.7 (6)	C22—N2—C18—C17	179.5 (3)
C4—C5—C6—C9	178.4 (4)	Cu1—N2—C18—C17	−0.3 (4)
C3—C2—C7—C6	−1.7 (6)	N1—C17—C18—N2	1.5 (4)
C1—C2—C7—C6	177.4 (4)	C16—C17—C18—N2	−178.5 (3)
C5—C6—C7—C2	2.8 (6)	N1—C17—C18—C19	−178.9 (3)
C9—C6—C7—C2	−177.3 (4)	C16—C17—C18—C19	1.1 (5)
Cu1^ii^—O3—C8—O4	−22.7 (4)	N2—C18—C19—C20	0.4 (5)
Cu1^ii^—O3—C8—C4	155.6 (2)	C17—C18—C19—C20	−179.2 (3)
C5—C4—C8—O4	−3.7 (5)	C18—C19—C20—C21	−0.8 (6)
C3—C4—C8—O4	176.1 (3)	C19—C20—C21—C22	0.9 (6)
C5—C4—C8—O3	178.0 (3)	C18—N2—C22—C21	0.2 (5)
C3—C4—C8—O3	−2.2 (5)	Cu1—N2—C22—C21	180.0 (3)
C5—C4—C8—Cu1^ii^	−132.2 (6)	C20—C21—C22—N2	−0.6 (6)

Symmetry codes: (i) *x*, −*y*+3/2, *z*+1/2; (ii) *x*, −*y*+3/2, *z*−1/2.
